# Sustainable and High-Performance Food-Packaging Films from Poly(butylene 2,5-furanoate) and Poly(pentamethylene 2,5-furanoate) Blends

**DOI:** 10.3390/polym18111372

**Published:** 2026-05-31

**Authors:** Arianna Palumbo, Michelina Soccio, Valentina Siracusa, Elisabetta Salatelli, Giulia Guidotti, Nadia Lotti

**Affiliations:** 1 Civil, Chemical, Environmental and Materials Engineering Department, University of Bologna, 40131 Bologna, Italy; arianna.palumbo3@unibo.it (A.P.); m.soccio@unibo.it (M.S.); nadia.lotti@unibo.it (N.L.); 2 Department of Chemical Science, University of Catania, 95125 Catania, Italy; vsiracus@dmfci.unict.it; 3 Department of Industrial Chemistry “Toso Montanari”, University of Bologna, 40136 Bologna, Italy; elisabetta.salatelli@unibo.it; 4 Interdepartmental Center for Industrial Agro-Food Research, CIRI-AGRO, Via Q. Bucci 336, 47521 Cesena, Italy

**Keywords:** bio-based polymers, furan-based polyesters, physical blends, barrier properties, flexible food packaging

## Abstract

Present research is focused on the preparation and characterization of bio-based polymer blends intended for sustainable food-packaging applications, starting from poly(butylene 2,5-furanoate) (PBF), characterized by very good barrier performance but quite high mechanical rigidity. In order to further improve gas permeability and increase its ductility, binary blends were prepared, combining PBF with varying amounts of poly(pentamethylene furanoate) (PPeF), another furan-based polyester with outstanding mechanical flexibility and gas barrier properties. The resulting materials were processed into compression-molded films and investigated through molecular, morphological, structural, thermal, and mechanical analyses. Blending turned out to be the winning tool in order to keep the high thermal stability of the reference homopolymers, increasing, at the same time, mechanical ductility and further lowering the permeability to oxygen and carbon dioxide compared to those measured for neat PBF. All these results were achieved without the use of any compatibilizer. Lastly, in order to test the end of life of these materials, composting studies were carried out, revealing a higher degree of weight loss for the blends compared with PBF homopolymer.

## 1. Introduction

The widespread use of plastic materials, the consumption of fossil sources for their production and the proper management of the resulting waste have become, at present, the main issues in terms of environmental sustainability. On the other side, their low cost compared to other classes of materials, high resistance and the wide range of obtainable properties make plastics a necessary component of everyday life. The market data confirm this assumption, as the global production reached approximately 430 million t in 2024, with more than 54 million t allocated to the European market [1].

In order to face the problem of plastic pollution, and considering that a world without plastic is almost unthinkable, many actions have been put in place, including policies aiming to drastically reduce pollution and greenhouse gases emissions, actions focused on achieving circular economy, and climate neutrality [2,3]. In this view, key strategies include improving waste management, enhancing reuse and recycling practices, and transitioning toward renewable feedstocks. As regards this last point, bio-based monomers, combined with environmentally friendly synthetic routes, offer a promising pathway toward more sustainable materials and the preservation of fossil feedstocks [4,5]. Additionally, the adoption of flexible packaging, which requires less material and energy compared to rigid formats both during production and transportation, can further contribute to reducing environmental impact [6]. All these actions will be even more impactful in the packaging sector, as the global market for plastic packaging was estimated at USD 480.62 billion in 2025 and is expected to reach approximately USD 835.88 billion by 2034, reflecting a compound annual growth rate of 6.41% over the forecast period [7]. As an example, within the European Union alone, replacing all food packaging with flexible alternatives could result in savings of approximately 26 million t of materials. Moreover, even in the absence of recycling, this shift would lead to a 40% reduction in the overall carbon footprint of packaging, corresponding to roughly 1% of total EU greenhouse gas emissions [8]. Lastly, although recycling represents a viable route to reduce the use of virgin materials, it is often limited by technical and economic constraints, especially for multilayer systems or food-contaminated packaging [9,10]. In addition, many developing regions lack the adequate infrastructure for efficient recycling, making landfilling still the dominant disposal option [11].

In this context, present research focuses on the development of novel bio-based polymeric blends with suitable mechanical and barrier properties for flexible food-packaging applications. The materials investigated belong to the family of aromatic polyesters, which are particularly attractive due to their high thermal stability, mechanical strength and barrier performance. More in detail, polymers derived from 2,5-furandicarboxylic acid (FDCA) have been selected, as this monomer can be entirely obtained from renewable feedstocks [12,13,14,15,16], and furan-based polyesters are increasingly being considered a sustainable alternative to terephthalic acid-based ones [17,18], all derived from fossil sources. Accordingly, FDCA has emerged as a highly promising platform chemical in recent years, with its market size, estimated at USD 1893.75 million in 2025, projected to grow at an annual rate of approximately 35% over the period of 2026–2034 [19]. This growth is also expected to contribute to a reduction in the production costs of this monomer, thereby facilitating the development of economically competitive packaging solutions. Moreover, compared to terephthalate-based polyesters and other materials commonly used for packaging applications, such as polyolefins and, among bioplastics, PLA, furan-based polyesters exhibit superior functional properties, particularly in terms of mechanical and barrier performance [18,20,21,22]. Notably, high-barrier food packaging can significantly extend the shelf life of food products, thereby contributing to the reduction in food waste, a critical issue with substantial economic and ethical implications.

More in detail, this research comes from the findings of a previous work from the authors [23], in which a physical blending procedure was applied, starting from poly(pentamethylene 2,5-furanoate) PPeF and poly(hexamethylene 2,5-furanoate) PHF, two homopolymers differing in only one methylene group in the glycolic subunit (5 for 1,5-pentanediol and 6 for 1,6-hexanediol). Through a simple, rapid, and industrially scalable processing technique [24,25], physical blends with intermediate mechanical properties compared to reference homopolymers, as well as barrier performance not particularly worsened compared to those of PHF, were obtained.

In order to improve the already good results obtained, in particular in terms of gas permeability—given the importance of this parameter for food-packaging purposes—in the present research, PPeF was mixed with poly(butylene 2,5-furanoate), PBF, another furan-based homopolymer which has attracted great interest in recent years, as documented by the many literature studies about its copolymers [26,27,28,29,30,31], blends [32,33,34] and composites [35,36,37]. PBF, which is made from FDCA and 1,4-butanediol, performs better than PHF, which contains a glycol with two additional methylene groups and is expected to increase the mechanical strength and resistance of PPeF while preserving its impressive gas permeability. Of note, compared to PHF, PBF is characterized by a lower degree of crystallinity [38], which is a very important feature in the case of furan-based polyesters. Indeed, for this class of materials a peculiar improvement of functional properties has been observed when the crystalline phase is suppressed and the glass transition temperature is close to the ambient one [38,39,40].

Last but not least, owing to the close chemical similarity of the constituent homopolymers, these blends can also be considered as systems approaching monomaterial characteristics, in view of a sustainable end of life. Indeed, high barrier performance is nowadays reached with multilayer packaging, the recycling of which is very difficult or not economically advantageous, due to the difficult separation of all the layers. The similar chemical structure would also ensure a good miscibility or at least compatibility between the two components of the blends, which, in turn, is a necessary requirement to obtain good processability and functional properties.

In detail, the two homopolymers, which were synthesized through two-step melt polycondensation, were then physically mixed in three different weight ratios (25:75, 50:50 and 75:25) and processed in compression-molded films. The films were then deeply characterized from the molecular, morphological, thermal, mechanical and surface-wettability point of view. Lastly, lab-scale composting tests were performed, and barrier properties to oxygen and carbon dioxide were evaluated to confirm their suitability as food-packaging materials.

## 2. Materials and Methods

### 2.1. Materials

Dimethyl 2,5-furandicarboxylate (DMF) was purchased from Sarchem Labs (Farmingdale, NJ, USA), 1,5-pentanediol (PeD) was purchased from Fluorochem (Glossop, UK), 1,4-butanediol (BD), and titanium tetrabutoxide (TBT), and titanium isopropoxide (TIP) were purchased from Sigma-Aldrich (Saint Louis, MO, USA) and were all reagent-grade.

### 2.2. Poly(butylene 2,5-furanoate) and Poly(pentamethylene 2,5-furanoate) Synthesis

Poly(butylene 2,5-furanoate) (PBF) and poly(pentamethylene 2,5-furanoate) (PPeF) were synthesized by two-step melt polycondensation, carried out in a 250 mL glass reactor. The system was continuously stirred at 100 rpm and maintained in a thermostatically controlled silicone oil bath, following the standard conditions commonly used for the synthesis of furan-based polyesters [38]. For PBF, the reagents used were 8.3 g (0.045 mol) of DMF, 12.2 g of BD (glycolic excess of 300 mol%, with respect to the diester), and 200 ppm each of TBT and TIP, used as catalysts, while for PPeF, the reagents used were 9.86 g (0.05357 mol) of DMF and 16.74 g of PeD (the same molar excess used for PBF), together with TBT and TIP in the same amounts as before. In the initial stage, performed at 190 °C under a continuous nitrogen flow, a transesterification reaction took place, with methanol progressively removed by distillation over approximately 90 min. The subsequent stage, which lasted about 2.5 h, was conducted at 200 °C under high vacuum (0.06 mbar) to remove glycolic excess and promote molecular weight growth, which was monitored by the increase in torque value. Once the torque reached a constant plateau, the reaction was terminated and the high-molecular-weight polyester was collected from the reactor.

### 2.3. Molecular Characterization

Proton nuclear magnetic resonance spectroscopy (^1^H-NMR) was carried out at room temperature to verify the chemical structure of PBF and PPeF, using a Varian Inova 400 MHz instrument (Agilent Technologies, Santa Clara, CA, USA). Before the analysis, 10 mg of each sample was solved in 700 μL of deuterated chloroform containing 0.03 vol % tetramethylsilane (TMS) as the internal standard. To completely solve PBF, a few additional drops of trifluoroacetic acid were added to the solution.

Gel permeation chromatography (GPC) was used to determine the molecular weights (M_n_) of the synthesized materials and the corresponding polydispersity indexes (D). Measurements were carried out at 30 °C using a 1525 binary HPLC pump (Waters, Milford, MA, USA) equipped with a PLgel 5 mm MiniMIX-C column (Agilent Technologies, Santa Clara, CA, USA). The samples were prepared by solving the homopolymers in HPLC-grade chloroform, the same solvent used as eluent, with a concentration of 2 mg/mL. In the case of PBF, few drops of hexafluoro isopropanol were added to completely solve the sample. To calculate the molecular weight values starting from the chromatograms, a calibration curve obtained from polystyrene standards in the range of 550–2,500,000 g/mol was used.

### 2.4. Blends Preparation and Processing

The two parent homopolymers were solved in the minimum weight amount of hexafluoro isopropanol/chloroform (5% *v*/*v*) and then mixed at room temperature and pressure under stirring in different weight amounts (75/25, 50/50 and 25/75). The resulting solutions were left under the hood for 24 h to allow for the complete evaporation of the solvent.

Films of the binary blends were made by compression molding, using a Carver C12 (Wabash, IN, USA) laboratory press. Approximately 3 g of each sample was heated in the press between two Teflon sheets to a temperature at least 20 °C above the melting temperature of PBF and left for 2 min in order to allow a complete melting. Afterwards, a pressure of 5 ton/m^2^ was applied for 1 min. Lastly, the obtained films were cooled to room temperature in the press. Prior to further characterization, all films were kept at room temperature for three weeks to allow them to reach the crystallization equilibrium.

### 2.5. Morphological Characterization

Scanning electron microscopy (SEM) was carried out on cryo-fractured cross-sections of each sample to evaluate the morphology and microstructure of the blends, as well as to evaluate the miscibility of the two reference homopolymers. An S-2400 scanning electron microscope (Hitachi Ltd., Tokyo, Japan) was employed, operating at 15 kV**.** Prior to the analysis, a gold conductive coating was deposited on each sample.

### 2.6. Thermal and Structural Characterization

TGA measurements were performed on the blends and the reference homopolymers by means of a Perkin Elmer TGA4000 (Waltham, MA, USA), under inert atmosphere (N_2_ flow of 40 mL/min). Measurements were carried out on approximately 5 mg of each sample, which was heated at 10 °C/min from 40 °C to 800 °C. T_onset_ was calculated as the point at which degradation begins, while T_max_ was calculated as the temperature corresponding to the minimum of the derivative thermogram.

Differential scanning calorimetry (DSC) was performed on the blends and the reference homopolymers by means a Perkin Elmer DSC6 (Waltham, MA, USA) under inert atmosphere (N_2_ flow of 20 mL/min), to ensure that the observed thermal transitions reflect the intrinsic behavior of the materials. Measurements were carried out on approximately 8 mg of each sample, which was subjected to the following thermal program: heating from −30 to 180 °C at 20 °C/min (I scan), rapid cooling from 180 to −30 °C at 100 °C/min, and further heating from −30 to 180 °C at 20 °C/min (II scan). From the calorimetric curves obtained, it was possible to calculate the glass transition temperature (T_g_) as the midpoint of the glass-to-rubber transition step, and the relative specific heat increment (ΔC_p_), as the height between the two baselines related to the glass-to-rubber transition step. The melting temperature (T_m_) and the cold crystallization temperature (T_cc_) were also calculated as the peak maximum/minimum of the endothermic/exothermic phenomena, respectively, together with the melting (ΔH_m_) and crystallization (ΔH_c_) enthalpies, from the corresponding areas of the peaks.

Wide-angle X-ray diffractometric (WAXD) measurements were performed on blend and homopolymers films by means of a PANalytical X’PertPro diffractometer (Almelo, The Netherlands) coupled with a copper source (λ = 0.154 nm) and equipped with a solid-state X’Celerator detector (0.1° steps, rate = 100 s/step). The degree of crystallinity (X_c_) was calculated as the ratio between the diffraction area of the crystalline portion and the total area of the diffraction profile.

### 2.7. Mechanical Characterization

Mechanical tests were carried out to determine the mechanical response of the materials under study upon tensile loading, by means of an Instron 5966 dynamometer (Norwood, MA, USA), equipped with a transducer-coupled 1 kN load cell. Films were cut in rectangular specimens (5 mm × 50 mm, gauge length of 20 mm), and stretched at a rate of 10 mm/min. The instrument records the applied load as a function of displacement and converts these data into stress–strain curves. The elastic modulus (E) was determined from the slope of the initial linear region of the curve, while the stress at break (σ_B_) and elongation at break (ε_B_) were obtained from the corresponding values at the point of failure. These values are reported as average value ± standard deviation calculated from measurements performed on six specimens for each material.

### 2.8. Gas Permeability Tests

In order to assess the barrier performances of the material under study, permeability to dry O_2_ and CO_2_ was evaluated on polymeric films (area of 78.5 cm^2^) using a manometric method with a GDP-C permeation-testing apparatus (Brugger Feinmechanik GmbH, Munchen, Germany), following the Gas Permeability Testing Manual and standards ASTM 1434-82 [41], DIN 53536 [42] and ISO/DIS 15105-1 [43]. The applied flow rate in the upper chamber of the instrument was 100 cm^3^/min, while a pressure transducer located in the lower chamber monitored the increase in gas pressure over time. The resulting pressure–time data were used to calculate the permeability (P) of the films located in between the two chambers.

### 2.9. Lab-Scale Composting Tests

The blends were subjected to lab-scale composting tests, to evaluate their degradation at different timepoints, from 1 up to 6 months. After being weighed, film specimens (1.5 × 1.5 cm) were placed between two layers of mature compost (kindly provided by “Nuova Geovis S.p.A”—HERA Group of Sant’Agata Bolognese, Bologna, Italy) and then incubated in a thermostatically controlled Julabo SW22 water bath (Seelbach, Germany). The system was kept at 58.0 °C and 90% relative humidity, under continuous agitation. Every month, sacrificial samples were removed from the compost, thoroughly washed with an aqueous solution containing 70 vol% of ethanol, and dried for at least 48 h. To determine if any degradation occurred, gravimetric weight loss was measured, SEM analysis was performed to observe the surfaces of the partially degraded samples, and lastly, DSC measurements were carried out on both the partially degraded films and the corresponding blanks (i.e., films incubated for 1 month at 58 °C under a humid environment, but without compost) to verify how the permanence in compost affected the crystallinity and the main thermal transitions of the materials.

## 3. Results and Discussion

In the present study, bio-based physical blends of PBF and PPeF were prepared and characterized for application in the field of sustainable, flexible food packaging. Of note, the two homopolymers are characterized by very similar chemical structure, differing just in one -CH_2_- group in the glycolic subunit. Therefore, good compatibility between the two reference homopolymers inside the blends is expected, and thus, an optimal performance of the final materials. Moreover, given the chemical structural similarity, the blend can be considered a monomaterial, thus presumably easy to recycle, also in view of a sustainable end of life.

### 3.1. Synthesis and Molecular Characterization

PBF and PPeF homopolymers were synthesized by two-step melt polycondensation using TBT and TIP as catalysts. The binary blends, designated as PBF_x_/PPeF_y_, where x and y represent the relative weight percentage of the two parent homopolymers, were prepared through the solubilization of different weigh ratios (25/75, 50/50 and 75/25) of PBF and PPeF in hexafluoro isopropanol/chloroform (5% *v*/*v*) under stirring and subsequent evaporation of the solvent. 

The homopolymers were first characterized from the molecular point of view by means of ^1^H-NMR spectroscopy, which allowed for the confirmation of their chemical structure and exclusion of the occurrence of side reactions during the polymerization process (Figure S1). More in detail, only the signals related to the polymers were detected: for both of the homopolymers, the singlet related to the furan subunit was located at δ 7.34 ppm for PBF and at δ 7.21 ppm for PPeF, respectively. Moreover, the signals of hydrogen atoms of 1,4-butylene moiety (4H, multiplet) and (4H, triplet) of PBF can be found at δ 1.86 ppm and δ 4.48 ppm, respectively, while for PPeF, the peaks due to the methylene protons of the glycolic subunit (2H, multiplet), (4H, multiplet) and (4H, triplet) were located at δ 1.55 ppm, δ 1.80 ppm, and δ 4.35 ppm, respectively.

GPC measurements were carried out to determine the molecular weights (M_n_) and the polydispersity index (D) of PBF and PPeF. The M_n_ values (Table 1) were high and comparable in both cases, together with polydispersity indexes in line with those obtained for polymers synthesized through polycondensation. The overall molecular characterization data allowed for the confirmation of the achievement of optimized polymerization conditions.

### 3.2. Morphological Characterization

**Figure 1:**
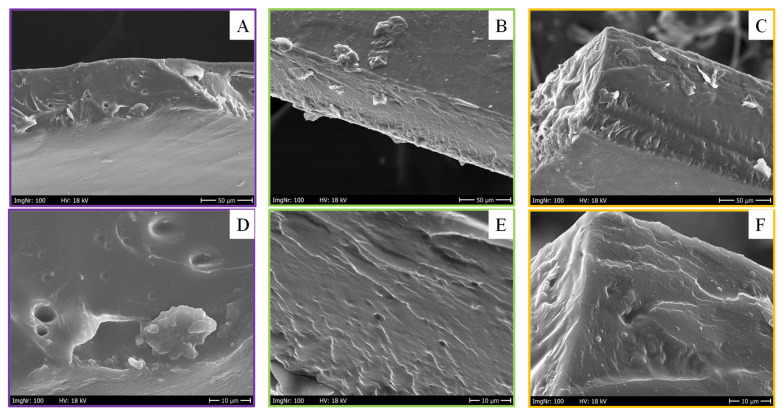
SEM pictures of cryo-fractured cross-sections at different magnifications (top 500×, bottom 2000×) of: (**A**,**D**) PBF_75_/PPeF_25_ (violet frame); (**B**,**E**) PBF_50_/PPeF_50_ (green frame); and (**C**,**F**) PBF_25_/PPeF_75_ (yellow frame).

To gain deeper insight into the morphology of the PBF_x_/PPeF_y_ blends, SEM micrographs of the film cross-sections at different magnifications are presented in Figure 1. These images provide valuable information regarding the morphological features of the blends and the possible presence of phase separation. As can be seen for all the samples evaluated, smooth and continuous fracture surfaces were obtained, indicating the two components are well-dispersed within each other. If pictures at higher magnifications (Figure 1D–F) are considered, only very few and small protrusions can be observed, especially in the mixtures richer in PBF, which should probably not impact the final material properties.

### 3.3. Thermal and Structural Characterization

**Figure 2:**
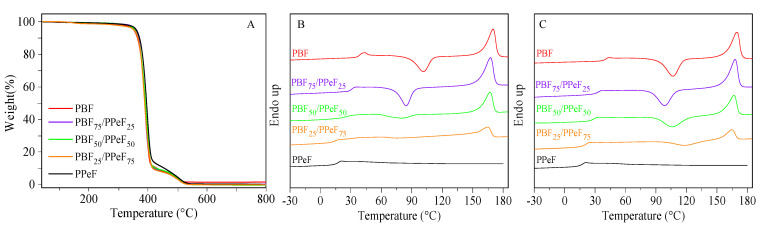
TGA curves. (**A**) The first (**B**) and the second (**C**) DSC scans of PBF, PPeF and PBF_x_/PPeF_y_ blends.

Three weeks after the molding, the films were subjected to deep thermal characterization. Thermogravimetric analysis (TGA) under inert atmosphere was first carried out to assess the thermal stability, a parameter of particular relevance for processing conditions. The obtained thermograms are shown in Figure 2A, while the extrapolated temperatures corresponding to the initial degradation (T_onset_) and to the maximum degradation rate (T_max_) are reported in Table 1.

According to the values obtained (T_onset_ > 370 °C and T_max_ ≥ 390 °C), a very high and similar thermal stability for PBF, PPeF and the resulting blends can be confirmed, suggesting that the physical mixing did not alter the already high stability of the starting homopolymers. This result is also in agreement with the previous literature studies on furan-based polyesters [38,39] and mixtures [23,44]. The obtained values are also higher than those measured for LDPE [45] and for commercially available bioplastics, such as poly(butylene succinate) (PBS) and polylactic acid (PLA) [46,47], and quite comparable to the values obtained for HDPE [45] and PET [48].

Lastly, a two-step degradation profile was observed for all the samples, with complete degradation at the end of the heating ramp.

Thermal transitions were measured by DSC analysis, which was carried out under inert atmosphere on film samples. From the first heating DSC scan (Figure 2B and Table 1), it is clear that the two reference homopolymers display the same phase behavior. Indeed, both are in a completely amorphous state, even though PBF is in a glassy amorphous phase, exhibiting the glass transition step above room temperature (37 °C), while PPeF is in a rubbery amorphous phase, with the corresponding DSC trace showing the endothermic baseline jump related to the glass transition below room temperature. Moreover, the PBF DSC trace shows an exothermic crystallization peak around 100 °C, and an endothermic melting peak at 170 °C. Since the crystallization enthalpy (ΔH_cc_) is very similar to the melting one (ΔH_m_), the PBF film can be considered practically amorphous.

The blends, on the contrary, are semicrystalline with a content of crystalline phase changing with composition. This result is quite surprising, considering the amorphous nature of both homopolymer films. The melting peak position suggests the crystalline lattice developed in the blends comes from the PBF fraction. The increase in PBF crystallization ability indicates the intimate mixing of the two polyesters (as observed in the SEM images in Figure 1) and, as a consequence, PBF glassy chains acquire higher mobility from the surrounding PPeF rubbery macromolecules. Moreover, the T_m_ values are slightly lower than that of PBF (in the range of 165–167 °C), suggesting partial miscibility between the two homopolymers. The PBF_75_/PPeF_25_ is the least crystalline with a very low crystallinity degree, due to a T_g_ value still above room temperature. In the PBF_50_/PPeF_50_ and PBF_25_/PPeF_75_ samples, the crystallization ability increases due to the increased chain mobility imparted by PPeF, which favors the chain folding of PBF chains. As a result, the blend with the highest PPeF content exhibits greater crystallinity than the one richest in PBF (Table 1), despite the fact that the developing crystalline phase is that of the PBF component.

Following the first scan, a second heating ramp was conducted after rapidly cooling the polymer melt to minimize crystallization phenomena and better observe the glass transition. Indeed, according to the literature [49,50], crystalline domains act as physical constraints, causing an increase in T_g_ values. The corresponding curves are shown in Figure 2C. The homopolymers exhibit behavior similar to that observed in the first scan. As regards the blends, these all show a single glass-transition step, further confirming the miscibility of PBF and PPeF, with T_g_ values being intermediate between those of the reference homopolymers and progressively decreasing with increasing PPeF content. Although all the blends undergo crystallization during scanning, they show a different crystallization capability: First, as the PPeF content increased, the macromolecular chains were able to crystallize during the heating scan at progressively higher temperatures, suggesting that the presence of mobile amorphous PPeF interferes with the crystallization process of the PBF-rich phase. Moreover, PBF_75_/PPeF_25_ and PBF_50_/PPeF_50_ blends are still semicrystalline (ΔH_cc_ < ΔH_m_), while the PBF_25_/PPeF_75_ blend appears completely amorphous (ΔH_cc_ = ΔH_m_), indicating that the presence of a high amount of non-crystallizable PPeF component completely hinders the crystallization of PBF chains during cooling.

The wide-angle X-ray diffractometric (WAXD) patterns of the tested samples are presented in Figure S2. As regards the homopolymers, no reflections due to the presence of a crystalline phase were observed in the PBF and PPeF WAXD spectra, which both show only a bell-shaped profile typical of fully amorphous material. As regards the blends, the one richest in PBF is characterized by a WAXD profile with a shoulder at 2θ 25.2°, while the diffractometric profiles of the other two compositions show two reflections of low intensity at 2θ 18.2° and 25.2°, respectively, a shoulder at 22.7°, and a very small peak at 10.7°, proving that the typical crystalline phase of PBF developed in these samples. Such profiles are very similar to those reported in the literature for pure semicrystalline PBF [27,51]. These reflections become more evident in the blends richer in PPeF, in line with the calculated X_c_ values and the calorimetric results (Table 1).

### 3.4. Mechanical Properties

**Figure 3:**
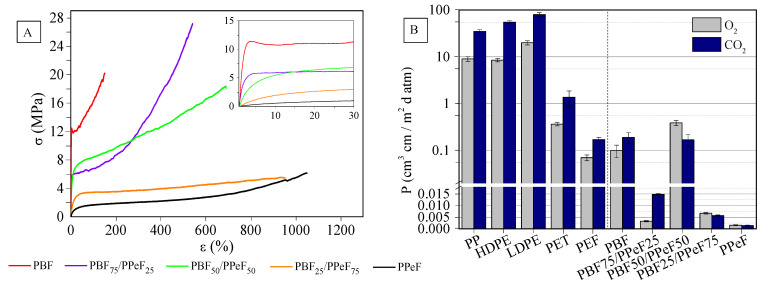
Mechanical and barrier performances of PBF, PPeF and PBF_x_/PPeF_y_ blends: representative stress–strain curves with a magnification of the initial elastic region (**A**); permeability values together with those of some commonly used packaging materials [58] and of PEF [59,60] (**B**).

To provide deep insight into the mechanical properties of the materials under investigation, and to check if the presence of PPeF is able to improve the performance of PBF, stress–strain measurements were carried out. The calculated tensile testing data (elastic modulus E, stress at break σ_B_ and strain at break ε_B_) are collected in Table 2, while stress–strain curves are shown in Figure 3A.

Taking into account that chain flexibility (i.e., T_g_ value) and degree of crystallinity are among the main factors influencing the mechanical behavior of polymers [52,53], it can be noted that PBF, which at room temperature is amorphous and glassy, is characterized by the highest elastic modulus, above 1000 MPa and a moderate elongation at break, around 150%. On the other hand, PPeF, amorphous and rubbery, is the polymer with the lowest elastic modulus at around 9 MPa and the highest elongation at break, exceeding 1000%, together with an almost immediate return to the original shape, indicating elastomeric behavior. It is interesting to note that this homopolymer exhibits peculiar behavior, as it can be processed into a self-standing film with exceptional elastic return [39]. This mechanical behavior has been attributed, as already discussed in the literature [39,40], to a peculiar arrangement in the material, responsible for its excellent functional properties, including mechanical ones.

As to the physical blends, the introduction of increasing amounts of PPeF leads to a progressive decrease in the elastic modulus (by approximately 3.5, 9, and over 40 times compared to PBF, respectively) and a parallel increase in the elongation at break, which improves by a factor of 3.5, 4, and 5.4, respectively (Table 2). The stress at break remains similar for all the samples except for the PBF_25_/PPeF_75_ blend, in which a slightly lower value close to the one of PPeF was obtained. Of particular importance is the fact that no yield was revealed for the blends, contrary to what was observed for the PBF homopolymer, indicating that small amounts of PPeF are sufficient to obtain blends with potential elastomeric behavior. Lastly, it is possible to notice a remarkable increase in toughness, once again due to the presence of PPeF (Figure 3A). This improvement is impressive, considering the fact that the two parent homopolymers are combined through simple physical blending, without the formation of chemical bonds between them. Furthermore, previous studies on furan-based blends have reported elongation at break values only slightly higher than those of the stiffest component in the binary system [54,55]. Therefore, the presence of good compatibility and strong interfacial cohesion between the two phases was confirmed, in agreement with the morphological observations (Figure 1). This behavior is also in line with thermal analysis (Table 1), as the improvement of mechanical flexibility can be related to the decrease in the T_g_ values by increasing the amount of PPeF in the blend. This effect also prevails in the development of a certain amount of crystalline phase (higher for the blends richest in PPeF homopolymer), which, on the contrary, usually induces a more brittle mechanical response.

Overall, these findings demonstrate that key mechanical properties, including flexibility and toughness, can be effectively tuned by simply adjusting the blend composition, making these materials well-suited for flexible packaging applications. Of note is the fact that this easy tunability allows the mechanical performance of the blends to match those of plastic materials currently used in flexible packaging applications [45]; notably, when compared to bioplastics such as PBS and PLA, the performance is highly superior [56,57].

### 3.5. Gas Barrier Properties

The permeability tests to oxygen and carbon dioxide were carried out on the compression molded films of all materials under study, to confirm their suitability as food-packaging materials. The corresponding permeability values are listed in Table 2 and shown in Figure 3B, where permeability values of some commercial plastics [58] and of PEF [59,60] are also reported.

All the materials investigated in this study exhibit outstanding barrier performance, surpassing those of PET [58] and approaching the values reported for PEF [59,60] for both O_2_ and CO_2_. To explain this remarkable behavior, it must be considered that, from a barrier point of view, the glassy amorphous phase usually limits chain mobility, thereby reducing the free volume available for gas diffusion. At the same time, crystalline domains further hinder gas transport due to their dense chain packing [61]. In the case of polymers containing furan rings, in addition to the former two phases, a further one—continuous and very dense, even if not ordered—is developed, with intermolecular pseudo-hydrogen bonding and aromatic π-π interactions being responsible for the formation of such a phase. This peculiar phase is even more effective than crystalline regions in limiting gas permeation, but its fraction is reduced by the formation of crystalline domains. The formation of one phase indeed competes with the formation of the other [38,62,63]. Last but not least, recent studies of some of the authors of the present paper indicated that the development of dense and continuous phase is favored when the T_g_ of material is at room temperature, since the macromolecular motions/rotations are unlocked, and thus macromolecular chains adjust their conformation, optimizing the amount of hydrogen bond-like and π-π interactions [64,65]. As a result, if the two homopolymers are considered, PPeF, which is rubbery and amorphous under the testing conditions, performs far better than PBF, which is amorphous but glassy. Observing the PBF/PPeF blends, a clear improvement can be noted upon the addition of 25 wt% of PPeF. This effect is attributable to the improved mobility of the whole amorphous phase (lower T_g_), being the two components miscible, compared to the PBF homopolymer (Table 1). On the contrary, the presence of 50 wt% of PPeF fraction leads to a detriment in the barrier performance of the PBF_50_/PPeF_50_ blend. This result may be due to the higher degree of crystallinity and therefore a higher percentage of interphases between the amorphous phase and the crystalline one, through which gas passage is favored. Lastly, permeability decreases again in the blend richest in PPeF, which is the best-performing material among the formulations, with values close to those of its reference homopolymer. Indeed, although semicrystalline, this sample contains the largest amount of highly performing PPeF. All these results further confirm the very good compatibility between the two homopolymers.

### 3.6. Lab-Scale Composting Studies

**Figure 4:**
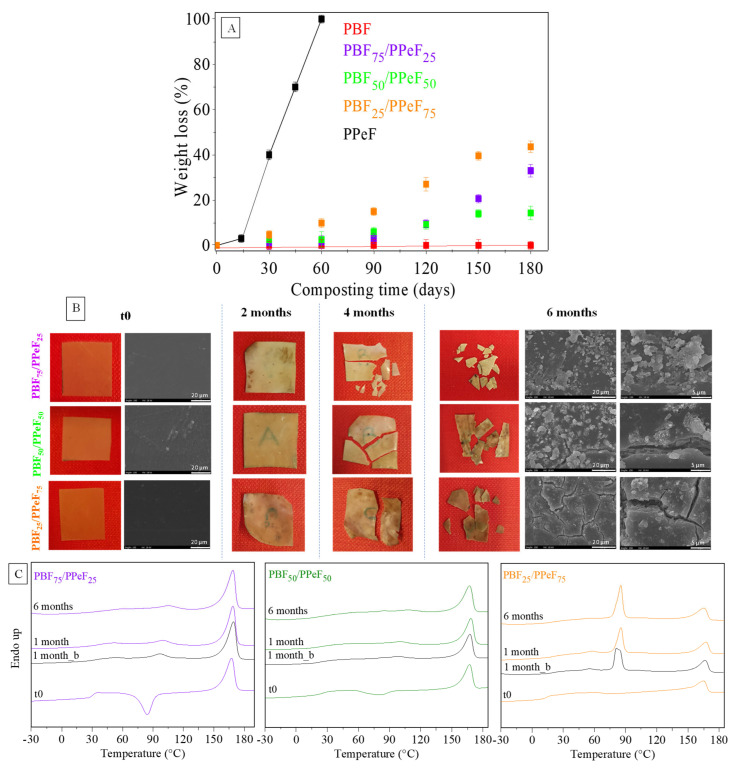
(**A**) Gravimetric weight loss as a function of incubation time of PBF, PPeF, and PBF_x_/PPeF_y_ blends. (**B**) Pictures and SEM images of neat (t0) and partially degraded PBF_x_/PPeF_y_ blends after incubation in compost. (**C**) I scan DSC profiles of partially degraded PBF_x_/PPeF_y_ blends after 1 and 6 months of incubation, together with those of relative blanks.

Compostability is a key property for materials used in short-life packaging, such as the food one. In such cases, compostable materials help reduce landfill accumulation, especially where recycling is impractical or inefficient. Previous studies carried out by some of the authors on both PBF and PPeF homopolymers already assessed the no-biodegradability trait in the composting of PBF, while in the case of PPeF, complete degradation occurred within two months of incubation [63]. The same lab-scale composting experiments were carried out on the blends under study, and the percentage weight loss as a function of composting time is shown in Figure 4A. A correlation between weight loss and the content of the degradable PPeF component can be observed, except for the PBF_50_/PPeF_50_ sample, which shows the lowest weight loss among the blends. One possible reason for this peculiar result may be the combined effect of a not-negligible crystallinity, higher than that of the blend richer in PBF (Table 1), and a relatively low amount of PPeF. Moreover, within the investigated timeframe, none of the samples underwent complete degradation. The maximum weight loss of approximately 45% after six months was recorded for the PBF_25_/PPeF_75_ blend.

As can be seen from the images shown in Figure 4B, the neat films are characterized by a smooth and quite transparent surface, while partially degraded samples become progressively more opaque and brittle, suggesting an increase in the degree of crystallinity. Furthermore, extensive fragmentation was observed, starting four months from incubation and becoming increasingly marked in the following timepoints.

The surface morphology of the films was also analyzed by SEM. As an example, Figure 4B shows images of the samples before and at the end of the composting experiment. As observed, the pristine films exhibit smooth and uniform surfaces, whereas significant morphological alterations occur after the composting test. Although complete weight loss is not achieved, pronounced surface defects—such as holes and cracks—become evident. These changes arise from the combined effects of bulk hydrolytic degradation and the enzymatic activity of microorganisms inside the compost, with the latter phenomenon predominantly occurring at the surface. More specifically, hydrolytic chain scission leads to the formation of cracks [64,66], while enzymatic degradation is responsible for the development of cavities [67,68]. As regards the blends under study, both enzymatic and hydrolytic degradation play a significant role: for example, in the PBF_75_/PPeF_25_ sample, circular agglomerates are mainly present, indicating the enzymatic activity is predominant, while cracks also form as the PPeF content increases, as proof that hydrolytic degradation takes place.

Lastly, to verify how the degradation process may have affected the thermal transitions of the materials under study, first-scan DSC analyses were conducted on the partially degraded films after 1 and 6 months of incubation and on blank samples, which were incubated for 1 month at 58 °C without compost. The DSC traces are shown in Figure 4C, while the corresponding thermal data are reported in Table S1. As a general trend, an increase in crystallinity can already be observed in all cases after one month of incubation: the crystallization peak disappears and only the melting one remains, in the same position with respect to the neat material, indicating that the main crystalline phase has not been affected. The fact that the DSC profiles of blanks and composted samples are very similar indicates that this result may mainly be due to the permanence at the incubation temperature. A slight further increase in crystallinity (higher ΔH_m_) between the samples after 1 and 6 months of incubation was observed (Table S1). Furthermore, in all cases, the presence of a new endothermic peak can be noted between 96 and 108 °C for the PBF_75_/PPeF_25_ and PBF_50_/PPeF_50_ blends and between 81 and 85 °C for the PBF_25_/PPeF_75_ sample, with this last peak being of much higher intensity. This phenomenon, which is related to the formation of a less perfect ordered phase than the higher-melting one, can be either due to annealing or to the development of PPeF crystalline phase [40]. Further studies will be carried out to verify the nature of this peak. Regarding glass transitions, no significant differences in the T_g_ value were found compared to the neat samples, except for PBF_25_/PPeF_75_ sample, for which T_g_ increased about 5 °C. In parallel, a decrease in Δc_p_ values was observed for all materials, indicating a progressive decrease in the amount of amorphous phase during the experiment, in agreement with the improvement of crystallinity.

## 4. Conclusions

In the present study, the reference furan-based homopolymer poly(butylene 2,5-furanoate) (PBF) was successfully physically mixed with varying weight amounts (25, 50 and 75 wt%) of poly(pentamethylene 2,5-furanoate) (PPeF), another furan-based polyester with a very similar chemical structure, into blends showing good compatibility between the two homopolymers, but also with very different solid-state properties. Indeed, PBF is a glassy material with good gas barrier performance but quite stiff behavior, while rubbery PPeF is a thermoplastic elastomer characterized by outstanding permeability to oxygen and carbon dioxide. According to the results obtained, a successful improvement of PBF properties was obtained through a very straightforward, rapid, and readily scalable approach. First, blending did not adversely affect thermal stability, which remains a key advantage of furan-based polymers. From a mechanical point of view, increasing the PPeF content leads to a progressive reduction in PBF stiffness, together with a marked increase in elongation at break of the final materials, which also showed no yield typical of PPeF and were all tougher than PBF. The gas barrier properties of the blends were superior to those of conventional packaging materials and also of PBF. Such an outstanding behavior has been explained as due to the good miscibility between the homopolymers, which form a microscopically dense structure particularly effective in hampering gas passage, despite the absence of covalent interactions between their macromolecular chains or the use of any compatibilizer. This hypothesis was also supported by SEM analysis. Lastly, composting tests revealed substantial fragmentation and enhanced degradability compared to PBF. It must be stated again that, although complete compostability was not reached, the similar chemical structure of the reference homopolymers allow for the consideration of the blends as if they were monomaterials and therefore easy to recycle, in view of a sustainable end of life.

In conclusion, the choice of the proper parent homopolymers, together with an easy physical modification approach, was very effective in obtaining materials suitable for sustainable flexible food packaging.

## Figures and Tables

**Table 1:** Molecular (GPC), thermal (TGA and DSC) and structural (WAXS) characterization data of PBF, PPeF and PBF_x_/PPeF_y_ blends.

	GPC		TGA		DSC												WAXS
	M_n_ KDa	D -	T_onset_ °C	T_max_ °C	I Scan						II Scan						X_c_ %
					T_g_ °C	∆c_p_ J/g°C	T_cc_ °C	∆H_cc_ J/g	T_m_ °C	∆H_m_ J/g	T_g_ °C	∆c_p_ J/g°C	T_cc_ °C	∆H_cc_ J/g	T_m_ °C	∆H_m_ J/g	
PBF	27.3	2.3	372	393	37	0.178	101	31	170	33	37	0.344	107	33	170	34	0
PBF_75_/PPeF_25_	-	-	371	390	30	0.428	85	28	167	32	32	0.350	98	31	168	34	2 ± 1
PBF_50_/PPeF_50_	-	-	373	394	25	0.254	80	9	167	23	28	0.385	106	21	167	26	11 ± 2
PBF_25_/PPeF_75_	-	-	373	396	16	0.263	-	-	165	11	20	0.360	119	11	165	11	8 ± 2
PPeF	29.6	2.4	377	399	16	0.319	-	-	-	-	16	0.277	-	-	-	-	0

**Table 2:** Mechanical characterization data and permeability values of PBF, PPeF and PBF_x_/PPeF_y_ blends.

	Tensile Tests			Permeability Tests	
	E MPa	σ_B_ MPa	ε_B_ %	P-O_2_ cm^3^ cm/m^2^ d atm	P-CO_2_ cm^3^ cm/m^2^ d atm
PBF	1290 ± 140	21 ± 3	157 ± 10	0.10	0.19
PBF_75_/PPeF_25_	372 ± 51	24 ± 4	564 ± 53	0.0033	0.0148
PBF_50_/PPeF_50_	158 ± 14	18 ± 3	631 ± 58	0.387	0.169
PBF_25_/PPeF_75_	31 ± 3	5 ± 1	850 ± 110	0.0067	0.0057
PPeF	9 ± 1	6 ± 1	1050 ± 200	0.0016	0.0014

## Data Availability

The raw data supporting the conclusions of this article will be made available by the authors on request.

## Supplementary Materials

The following supporting information can be downloaded at https://www.mdpi.com/article/10.3390/polym18111372/s1, Figure S1: ^1^H-NMR spectra of PBF (top) and PPeF (bottom) homopolymers, with peaks attribution; Figure S2: WAXS profiles of PBF, PPeF and PBF_x_/PPeF_y_ blends; Table S1: Thermal (DSC) characterization data of partially degraded PBF_x_/PPeF_y_ blends after 1 and 6 months of incubation in compost at 58 °C, together with those of blanks after 1 month of incubation at 58 °C.
