# Supplementary material for: Sustainable and High-Performance Food-Packaging Films from Poly(butylene 2,5-furanoate) and Poly(pentamethylene 2,5-furanoate) Blends
Source: Polymers (Basel). 2026 May 31;18(11):1372. doi: 10.3390/polym18111372 (PMC13258956; doi:10.3390/polym18111372)
Supplement: Supplementary file 1 [file polymers-18-01372-s001.zip › polymers-4322318-supplementary.pdf]

Supplementary Information

Sustainable and high-performance food packaging films from poly(butylene 2,5-furanoate) and poly(pentamethylene 2,5-furanoate) blends

Arianna Palumbo[1], Michelina Soccio[1], Valentina Siracusa[2], Elisabetta Salatelli[3], Giulia Guidotti[1*], Nadia Lotti[1,5]

[1] *Civil, Chemical, Environmental and Materials Engineering Department, University of Bologna, 40131 Bologna, Italy;*

[2] *Department of Chemical Science, University of Catania, 95125 Catania, Italy;*

[3] *Department of Industrial Chemistry "Toso Montanari", University of Bologna, Viale Risorgimento 4, 40136 Bologna, Italy;*

[4] *Interdepartmental Center for Industrial Agro-Food Research, CIRI-AGRO, Via Q. Bucci 336, 47521 Cesena, Italy*

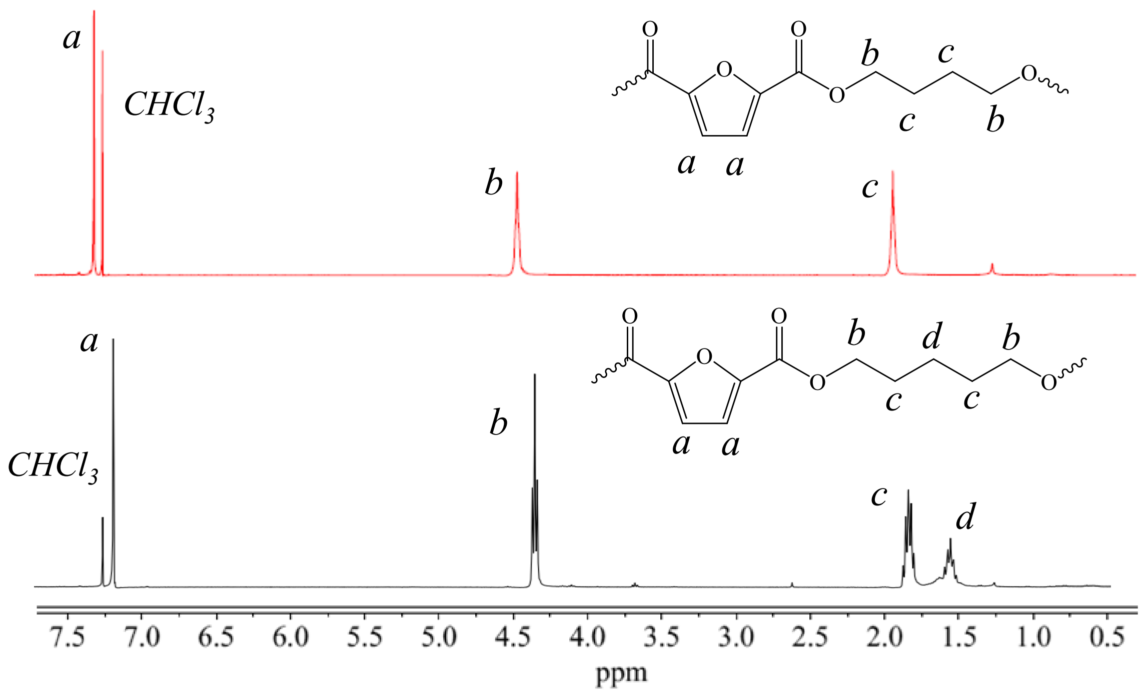

Figure S1. ^{1}H-NMR spectra of PBF (top) and PPeF (bottom) homopolymers, with peaks attribution.

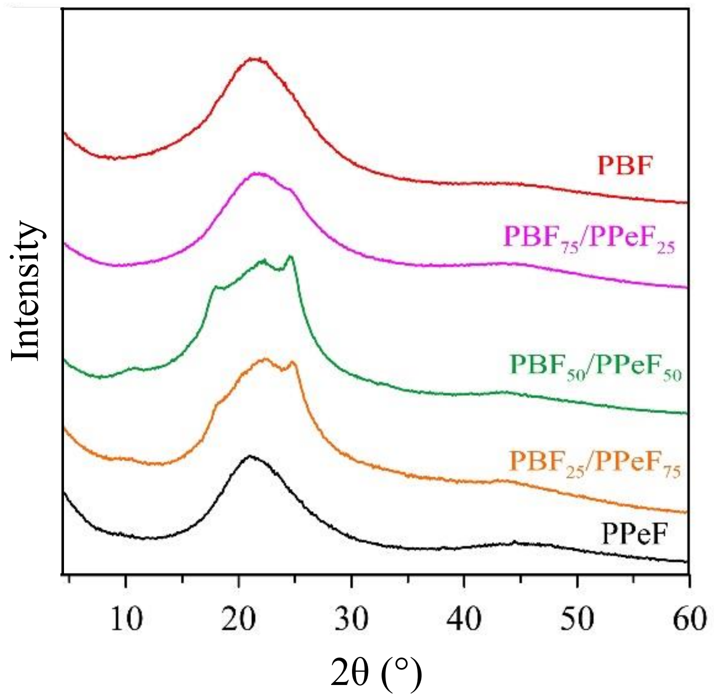

Figure S2. WAXS profiles of PBF, PPeF and $PBF_x/PPeF_y$ blends.

Table S1. Thermal (DSC) characterization data of partially degraded $PBF_x/PPeF_y$ blends after 1 and 6 months of incubation in compost at 58 °C, together with those of blanks after 1 month of incubation at 58 °C.

	**$PBF_{75}/PPeF_{25}$**				**$PBF_{50}/PPeF_{50}$**				**$PBF_{25}/PPeF_{75}$**			
	**t0**	**1 month**	**1 month_b**	**6 months**	**t0**	**1 month**	**1 month_b**	**6 months**	**t0**	**1 month**	**1 month_b**	**6 months**
**T_g °C**	30	34	32	34	25	26	24	n.d.	16	20	21	22
**ΔC_p J/g°C**	0.428	0.291	0.122	0.183	0.254	0.144	0.133	n.d.	0.263	0.189	0.199	0.193
**T_{cc} °C**	85	-	-	-	80	-	-	-	-	-	-	-
**ΔH_{cc} J/g**	28	-	-	-	9	-	-	-	-	-	-	-
**T_m °C**	167	100<br>169	96<br>169	106<br>169	167	101<br>168	98<br>168	108<br>167	165	85<br>167	81<br>167	85<br>165
**ΔH_m J/g**	32	5.2<br>38	5.0<br>38	5.1<br>42	23	2.8<br>26	2.6<br>24	2.2<br>29	11	15<br>13	16<br>13	20<br>14
